# G Protein-Coupled Estrogen Receptor: A Potential Therapeutic Target in Cancer

**DOI:** 10.3389/fendo.2019.00725

**Published:** 2019-10-25

**Authors:** Shen Xu, Shan Yu, Daming Dong, Leo Tsz On Lee

**Affiliations:** ^1^Department of Orthopedics, The First Affiliated Hospital of Harbin Medical University, Harbin, China; ^2^Faculty of Health Sciences, Centre of Reproduction Development and Aging, University of Macau, Macau, China; ^3^Cancer Centre, Faculty of Health Sciences, University of Macau, Macau, China

**Keywords:** G protein-coupled estrogen receptor, cancer, signaling pathway, estrogen, non-genomic estrogen pathways

## Abstract

The G protein-coupled estrogen receptor (GPER) is a seven-transmembrane-domain receptor that mediates non-genomic estrogen related signaling. After ligand activation, GPER triggers multiple downstream pathways that exert diverse biological effects on the regulation of cell growth, migration and programmed cell death in a variety of tissues. A significant correlation between GPER and the progression of multiple cancers has likewise been reported. Therefore, a better understanding of the role GPER plays in cancer biology may lead to the identification of novel therapeutic targets, especially among estrogen-related cancers. Here, we review cell signaling and detail the functions of GPER in malignancies.

## Introduction

Steroid hormones, such as estrogen, mediate important physiological effects via classical estrogen receptors (ER, including ERα and ERβ) (1). Upon ligand binding and dimerization, these receptors translocate into the nucleus and directly regulate specific target genes by binding estrogen responsive elements (ERE). The structure and functions of ERs have been elucidated in detail (2). Previous studies also reported that estrogens are involved in carcinogenesis via the regulation of apoptosis, cell proliferation and the cell cycle (3, 4). Apart from classic ER-dependent genomic regulation, a non-genomic pathway via which estrogen mediates cellular activities has been discovered, in which estrogens bind the G protein-coupled estrogen receptor (GPER) and directly trigger cellular signaling events.

GPER, also known as GPER1 or GPR30, was first discovered in 1996 in breast cancer tissue (5). Its cDNA sequence was cloned by Takada et al. in 1997 via differential cDNA library analysis of the human breast adenocarcinoma cell lines MCF7 and MDA-MB-231 (6, 7). The human GPER gene is located on chromosome 7 and contains an open reading frame with 1,128 bp that encodes a 375-amino acid receptor (5, 8). Expression of this receptor is significantly stronger in triple-negative MDA-MB-231 cells when compared to ER-positive MCF7 cells. In addition, GPER is strongly expressed in triple-negative breast cancer (TNBC) cells, including MB-468 and MDA-MB-436 (9). Thus, GPER likely plays a significant role in cancer biology via an ER-independent pathway. As this receptor is a member of the G protein-coupled receptor (GPCR) family and was discovered in breast cancer tissue, it was initially termed GPCR-Br. Shortly afterward, this receptor was considered to be an orphan receptor and was renamed GPR30. Since follow-up studies confirmed that this receptor is highly specific to E2 as well as related analogs (10) and mediates rapid non-genomic estrogen effects (7, 11, 12), the receptor has become more widely known as GPER.

GPER is expressed in a variety of tissues including the nervous, reproductive, digestive, and muscle apparatus (13). The expression of GPER was understood to be limited to the cell surface until recently; however, a number of studies have suggested that the binding domain of this receptor is also expressed on the endoplasmic reticulum (12, 14). In addition, GPER expression is independent to that of ERs. A GPER-lacZ reporter mouse revealed expression of GPER in gastric chief, small arterial endothelial and smooth muscle cells (15). Expression has also been reported in cerebral pericytes as well as cells of the cortex and dentate gyri, anterior pituitary and adrenal medulla (15).

Regarding the cellular localization of GPER, there is still under debate about the localization of GPER. Since it was classified as a member of GPCRs, discussion of the locations for this receptor is focused on the plasma membrane and early reports considered GPER with a cell membrane expression (16). However, more and more evidence revealed that the subcellular localization of GPER with endoplasmic reticulum and having the major functions intracellularly (17, 18). But the traffic for GPER to shift its location during the ligand-induced response from endoplasmic reticulum to plasma membrane or transportation inversely could not be neglected as this may indicate the GPER function in a cell-dependent manner. Meanwhile, the existence of estradiol binding entities both in the plasma membrane and the endoplasmic reticulum also support the intracellular function of GPER (19). Researchers also observed E2 could trigger the ion transportation and protein kinase activation without new protein production (20). This phenomenon was not explained until the discovery of ER isoforms and GPER in the endoplasmic reticulum. Those new understanding of ER-related signals suggested estradiol could also cause non-classical signals from the receptors located on the endoplasmic reticulum.

## GPER Agonism and Antagonism

The endogenous steroid hormone 17β-estradiol (E2) is a predominant ligand of GPER (21). Other 17β-estradiol-based steroids, such as estriol (E3), E3-sulfate (estriol-3-sulfate and estriol-17-sulfate), and estrone also interact with GPER (22). In contrast to the effects of these steroids on the ER, estrone and E3 serve as GPER antagonists (23). In addition, phytoestrogens (e.g., flavones, isoflavones, lignans, coumestans, saponins, and stilbenes) as well as synthetic estrogenic compounds within pesticides, herbicides and some plastic monomers (including bisphenols, alkylphenols, methoxychlor, polychlorinated biphenyls, and dioxins) also activate GPER (24, 25). Although such xenoestrogens are more potent in activating ERα and ERβ (26), some research suggests that the two major phytoestrogens, genistein and quercetin, also stimulate c-fos expression via an ER-independent manner (i.e., GPER) in ERβ-positive MCF7 and ERα-negative SKBR3 breast cancer cells (27).

A number of small molecules have been identified as agonists or antagonists specific to GPER and were not noted to interact with ERs. The GPER-selective agonist G-1 was identified in 2006 by Bologa et al. and was reported to exert no significant effects on other GPCRs or ERs (28, 29). Effects of G-1 in GPER knockout models were negligible (30), suggesting that G-1 is a specific agonist of GPER. In a mouse model of ischemia-reperfusion injury, G-1 significantly improved intestinal mucosal damage and alleviated neutrophil infiltration (31). Interestingly, an increase of E2 and G1 sensitivity in tamoxifen-resistant MCF-7 cells has been reported (32). Such findings indicate that blockage or desensitization of the ER pathway enhances the GPER response.

The selective antagonist G-15, identified in 2009, affects mouse uterine function *in vivo*. The E2-induced proliferation of uterine epithelia was reduced by ~50% after treatment with G-15 (33). Meso-octamethylcalix-[4]-pyrrole (C4PY), a GPER antagonist, was found to inhibit E2 and G-1 mediated *c-fos* activation and EGR1 promoter activities in SKBR3 breast cancer cells as well as cancer-associated fibroblasts (CAFs) (34). Another GPER antagonist, G-36, was synthesized by Dennis et al. in 2011. As G-36 was found to exhibit lower off-target activity and weaker cross-reactivity with ERα as compared to G-15, it has become widely used in the study of GPER (35). In addition, tamoxifen has been reported to act as a GPER agonist in SKBR3 cells (36, 37). ICI182,780 (fulvestrant), an ER antagonist (38), was likewise reported to be a GPER agonist (11).

The affinity of different agonists and antagonist to GPER have huge variations. For the well-known agonists G-1, it has Ki approximately 10 nM with GPER which is similar to E2 (Ki ~6 nM). G-1 has no binding toward either ERα or ERβ until concentrations more than 10 μM (33). Another agonist, ICI 182,780 (fulvestrant) with a binding affinity of 30–50 nM to GPER (39). For the xenoestrogens, BPA and dichlorodiphenyltrichloroethane (DDT) have relatively lower affinity to GPER, the affinity of BPA is about 0.6 mM. For DDT, depend on the isomer structure the affinity ranged from 2.8 to 10 mM (40). For antagonist G15, it has a 0.5-fold better binding compared with G-1, and 1,000-fold selectivity against for ERα and ERβ (33). Estriol (E3) also has a potential antagonist effect with GPER in SkBr3 cells and the cell proliferation induced by 100 nM G-1 can be abolished by 1 μM E3 (41). A radiolabeled synthetic antagonist, iodinated tetrahydroquinolines, demonstrated a selective binding with GPER at 20 nM range in human endometrial cancer Hec50 cells, which cell did not express ERs (42). Nayak et al. also found that the GPER binding affinity of ^113^In-G-DOTA is around 34 nM which is comparable to the similar G-1 (~11 nM) and G15 (~20 nM) by the ^111^In-labeled non-steroidal imaging receptor binding affinity assay Hec50 cells (43).

## Signaling Pathways of GPER

### G Protein-Dependent Pathway

As a classical GPCR, GPER was found to participate in G protein-dependent cell signaling. GPER activates the subunit of Gα, and subsequently adenylate cyclase (AC), to increase cAMP production that in turn leads to the activation of protein kinase A (PKA) and deactivation of *raf-1* (39, 44). However, the increase in cAMP accumulation also inhibits the production of matrix metalloproteinases (MMPs). Interestingly, prior research has reported massive G-1 induced tumor necrosis in prostate cancer, but aggravation of breast cancer progression (45, 46). Such contrasting effects underscore the bilateral role played by GPER in cancer biology. Estrogen-activated GPER was additionally found to activate the α5β1 integrin in a Gβγ-dependent pathway. Activation of α5β1, in turn, was reported to subsequently induce fibronectin matrix assembly and the release of growth factors in SKBR3 breast cancer cells (47). Activation of GPER also stimulates intracellular calcium mobilization, indicating that GPER activates the Gαq pathway. Such GPER-mediated calcium mobilization is completely blocked by the epidermal growth factor receptor (EGFR) inhibitor AG1478, but not by the PLC inhibitor U73122. These findings indicate that calcium mobilization is mediated by the transactivation of EGFR rather than the activation of the classical Gαq pathway (7).

### Transactivation of EGFR

As mentioned above, GPER transactivates EGFR, stimulating the associated downstream signaling pathway (10). Transactivation occurs via an EGFR ligand-dependent pathway; GPER increases MMP expression, thus stimulating the release of membrane-anchored EGFR ligands. In this pathway, the activation of GPER dissociates the G-βγ complex and activates the downstream Src-related tyrosine kinase family as well as phosphorylation of the Shc adapter protein, enhancing MMP expression. These events, in turn, cause the release of heparin-binding epidermal growth factor (HB-EGF) due to proteinase activity (46). The released EGF ligands subsequently activate EGFR and initiate the mitogen-activated protein kinase (MAPK)/phosphoinositide 3-kinase (PI3K) pathway (21), as well as the Akt pathway (48). Activation of EGFR also triggers the downstream Src kinase family to phosphorylate Raf, which in turn stimulates extracellular signal-regulated kinase (ERK) phosphorylation, and activation of a number of transcription factors including, c-Myc, c-fos, and c-jun (49).

### HIF Induced Pathway

Hypoxia-inducible factor (HIF) is closely related to GPER in cancer cells. Under hypoxia, activation of the HIF pathway also activates downstream GPER signals and protects cells from apoptosis (50). The HIF/GPER pathway was also found to upregulate vascular endothelial growth factor (VEGF) expression in CAFs (51). As the human GPER promoter contains several HRE consensus motifs, GPER expression can, therefore, be directly upregulated in the setting of hypoxia. Mimicry of hypoxic conditions utilizing CoCl_2_ was reported to upregulate GPER expression in ER-negative breast cancer cells via the HIF-1α dependent pathway (50). In addition, GPER was found to interact with HIF-1α to enhance CTGF, VEGF, and IL-6 expression in breast cancer cells and CAF (51, 52). Interestingly, HIF dimers were found to form a heterocomplex with GPER and bind hypoxia-responsive elements (HREs) under hypoxic conditions (53). Chromatin immunoprecipitation (ChIP) assay confirmed that HIF and GPER are recruited to the VEGF promoter (51). These findings suggest that GPER plays critical roles in hypoxia-related vasculogenesis and directly regulates gene expression by forming heterocomplexes with HIF.

### Notch Signal Pathway

Notch signaling is vital in cell-cell interactions; it plays an important role in the epithelial-mesenchymal transition (EMT) as well as the mediation of cancer cell and CAF responses to the microenvironment (54–56). As the ERα pathway is suppressed in the setting of hypoxia, GPER likely plays a more significant important role in the mediation of estrogen responses in such a setting. Both E2 and G-1 recruit the intracellular Notch-1 (N1ICD) domain to the Hes-1 promoter, activating reporter gene and Snail expression in SkBr3 cells (55). The migratory ability of SkBr3 cells thus becomes enhanced. Since this effect can be suppressed by a γ-secretase inhibitor (GSI) or dominant-negative-MAML-1, both Notch activation and Notch-dependent transcriptions are involved in mediating such E2 and G-1 enhanced cell migration. In addition, as G-1 can prevent the expression of vascular endothelial cadherin, GPER likely plays an important role in cancer metastasis.

### Insulin-Like Growth Factor Receptor (IGFR) Pathway

Interaction between IGF-I and the estrogen pathway is well-known to enhance cell proliferation and suppress apoptosis (57). Recent findings suggest that such cross-talk is not only limited to the classical nuclear receptor pathway, but also involves GPER. In MCF-7 breast cancer cells, insulin-like growth factor-1 (IGF-1) activates the GPER promoter and increases the GPER transcription through the IGF-IR/PKCδ/ERK pathway (58). Interestingly, IGF-1-mediated cell migration and proliferation of MCF-7 and endometrial (Ishikawa) cancer cells require GPER expression. Study of mesothelioma and lung cancer cells has also suggested that the collagen receptor discoidin domain 1 (DDR1)-mediated upregulation of connective tissue growth factor (CTGF) and early growth response 1 (EGR1) upon IGF-I stimulation requires both IGF-IR and GPER (59).

### NF-κB Pathway

Chemokine ligands are usually considered to be inflammatory factors, and research has suggested that the CXC motif chemokines are involved in GPER function. Luciferase reporter assay evaluating the NF-κB reporter found that GPER suppresses TNFα-mediated NF-κB promoter activity in a dose-dependent manner. In SKBR3 cells, G-1 was found to significantly inhibit the expression of interleukin-6 (IL-6); however, this could be reversed by NF-κB inhibition. The effect of GPER on IL-6 expression is thus dependent on NF-κB (60). A similar NF-κB-dependent pathway was identified in cadmium-induced thyroid cancer, in which GPER was found to upregulate cyclin A and D1, as well as secretion of IL-8 (61). In that pathway, the activation of GPER by cadmium, E2 or G1 was found to trigger NF-κB translocation via the ERK and Akt cascades and cause cell proliferation, invasion and migration of thyroid cancer cells (61).

### Hippo/YAP Pathway

Genes involved in the Yes-associated protein 1 (YAP)/PDZ-binding domain (TAZ) pathway, including cysteine-rich angiogenic inducer 61 (CYR61), endothelin 1 (EDN1), CTGF, and EGR1 were found to be regulated by GPER (62, 63). These findings shed new light concerning the role of GPER in the Hippo pathway and in the control of cancer cell proliferation. Activation of GPER was found to induce phosphorylation of YAP/TAZ via Gαq-11, PLCβ/PKC, and Rho/ROCK, enhancing the proliferation and migration of breast cancer cells. In addition, GPER and TAZ expression in invasive ductal carcinoma (IDC) are positively correlated; high expression of GPER may contribute to IDC initiation via the YAP/TAZ pathway. The Hippo pathway, therefore, is one of the key downstream GPER signaling pathways that regulate physiological function in breast tumorigenesis (64). In pancreatic cancer, the activation of GPER by tamoxifen alters the tumor microenvironment by Rho-A mediated YAP deactivation (65). This pathway allows tamoxifen to suppress myofibroblastic differentiation of pancreatic stellate cells (PSCs) and, in turn, promote cancer cell invasiveness.

### Cross-Talk With Other Receptors

The mineralocorticoid receptor (MR), also known as the aldosterone receptor was suggested to have cross-talk with GPER and affects the aldosterone function (66). Aldosterone, as an important therapeutic target of cardiovascular diseases, could partially be activated by MR-GPER cross-talk and triggers certain non-classical and non-genomic functions for the myocardium (67). *In vivo* experiment also suggested the inhibition of GPER could suppress murine renal cortical adenocarcinoma cell pulmonary metastatic cancer spread. This provided solid evidence that MR-aldosterone function on promoting renal cancer spread could be influenced by GPER. However, the detailed molecular mechanism about the MR-GPER cross-talk is still largely unknown (68).

More than the above-mentioned estrogen and MR pathways, the rapid non-genomic pathway related to other sex-related steroids (such as androgens and progestins) are also observed in regulating multiple biological processes. Those exciting results further extended our board knowledge into the hormonal therapy for the cancers. However, the complex regulatory networks also reflected by the controversial phenotypes in different cancers (69). As Migliaccio et al. pointed out, the major problem is due to the lack of any model with “ONLY” non-genomic receptor actions. Also, the role of the rapid pathways in hormonal therapy resistance and the potential cross-talk between estrogen and those steroid receptors are still waiting for intensive investigations (70).

## The Role of GPER in Cancer Cells

Since the discovery of GPER in breast cancer, its role in malignancies has been of great interest. As estrogen is deeply involved in cancer progression and metastasis, non-genomic estrogen effects exerted by GPER has become an interesting topic of study, aiming to further detail ER-negative cancer cell properties. Below, we discuss the roles GPER plays in various cancers.

### Breast Cancer

The involvement of GPER in breast cancer progression and metastasis was reported in many publications. In the majority of breast cancer cells, GPER is highly expressed when compared with normal tissues (71). More importantly, high GPER expression has been found to strongly correlate with a poor prognosis (72). In ER-positive breast tumors, the most common pathologic subtype, GPER expression was found to correlate with shorter overall patient survival time (73). In the ER-positive MCF-7 cancer cell line, GPER agonists were found to induce the ERK1/2 pathway (74). Ahola et al. suggested that overexpression of GPER enhances tamoxifen-induced cancer apoptosis via the PI3K/MAPK/STAT pathway (75). Interestingly, tamoxifen was found to stimulate the mRNA expression of GPER, which in turn transactivates the EGFR signaling pathway (7). The SKBR3 (GPER and HER2 positive, ER-negative) cell line is widely used to study the effects of GPER (76). As discussed above, EGFR transactivation by GPER occurs by the ligand-dependent pathway, thereby promoting SKBR3 cell proliferation (39). Because EGF also upregulates GPER expression, this positive feedback loop is thus a critical mechanism for tamoxifen resistance (77). Moreover, bisphenol A hexafluoride (BPAF), previously described to be representative of environmental endocrine disruptors, has also been reported to expedite the initial rate of cell proliferation and migration (78). Although BPAF-mediated gene transcription can be inhibited by GPER knockdown in the ER-positive T47D breast cancer cell line (79), the mechanisms of how tamoxifen improves breast cancer patient outcomes remain unclear. Further investigations are required to detail the interrelationship between GPER and ER in breast cancer cell apoptosis.

In breast cancer cell lines, MDA-MB 231 and SUM159, the interaction between the focal adhesion kinase (FAK) and GPER was found to take a major function in cancer cell migration, adhesion and invasion (80). Bioinformatics analysis from The Cancer Genome Atlas (TCGA) also suggested a higher PTK2 gene level (gene encoding FAK) was found in TNBC samples. Further investigations revealed that estrogens through GPER triggered Y397-FAK phosphorylation and increased focal adhesion points. Inhibition of FAK suppresses the estrogens/GPER mediated cell migration in MDA-MB 231 and SUM159 cells. The activation of FAK is through the GPER/c-Src/MEK transduction pathway. Xenoestrogens, BPA also activates FAK via GPER pathway in MDA-MB-231 cells (81). Moreover, in TNBC cells, the GPER-FAK transduction pathway is considered in more comprehensive targeted therapies and validated.

Interestingly, another recently published research highlighted that androgens binding with classical AR has almost the same effect of GPER in Src/PI3K/FAK pathway to enhance the cancer cell invasive probabilities in TNBC cells (82). This phenomenon strongly implied other sex steroids had shared the pathways in extranuclear rapid non-genomic signaling networks as ER and PR levels are almost undetectable in TNBC cells.

Due to ER and HER2 deficiency, TNBC treatment still relies on cytotoxic chemotherapy, including poly ADP-ribose polymerase (PARP) inhibition, angiogenesis, and EGFR (83). Although patients with TNBC respond well to such therapies, they suffer higher risks of relapse and worse disease progression after chemotherapy to the TNBCs (84). Since TNBC tumors exhibit high levels of GPER (85), their resistance to classic hormonal therapy is likely closely linked to this receptor (86). Upon GPER knockdown, TNBC cell proliferation also slowed due to reduced EGFR and c-fos activation (87). Hence, GPER is a potential novel therapeutic target in the treatment of TNBC.

Interaction between Na^+^/H^+^ exchanger regulatory factor 1 (NHERF1) and GPER have been demonstrated in MDA-MB-231 cells by co-immunoprecipitation and co-immunofluorescence staining. Overexpression of NHERF1 inhibits the phosphorylation of ERK1/2 and Akt via the GPER related pathway and finally inhibits triple-negative breast cancer cell proliferation (88). Study of GPER knockout mice has suggested that although GPER does not influence breast cancer tumorigenesis, it is likely involved in cell differentiation and migration. No differences between knockout and wild type mice were noted in regard to tumorigenesis. However, tumors in knockout models were smaller in size and less proliferative than in wild-type controls (89). Moreover, GPER was also found to affect the breast tumor microenvironment. Juan et al. suggested that activation of GPER increases CAF cell proliferation under hypoxic conditions (52). GPER was reported to upregulate IL-6, VEGF and CTGF expression in CAF. Indeed, GPER levels were proposed to be a marker of breast cancer aggressiveness (90), but further investigation including *in vivo* study and clinical analysis is required to confirm this hypothesis.

### Ovarian Cancer

Satoe Fujiwara et al. reported that high expression of GPER is associated with a poor outcome in ovarian cancer patients; GPER was found to enhance the phosphorylation of Akt via EGFR transactivation, subsequently enhancing ovarian cancer cell proliferation (48). A similar study by Heublein et al. investigated follicle stimulating hormone receptor- (FSHR) and luteinizing hormone receptor- (LHCGR) negative epithelial ovarian cancer patients. In those patients, lower GPER expression was found to be positively associated with overall survival time (91). Ignatov et al. controversially reported that high GPER expression was a positive factor influencing 2-year disease-free survival in ovarian cancer patients. That *in vitro* study suggested that GPER is responsible for a significant increase in cellular apoptosis and cell cycle arrest in SKOV-3 and OVCAR-3 cells (92). Another study that investigated IGROV-1 and SKOV-3 cell lines also reported that G-1 suppresses cellular proliferation and induces apoptosis by targeting tubulin (93).

### Cervical Cancer

The role of estrogen in the pathogenesis of cervical cancer has been widely studied. Using human papillomavirus-transgenic mice, Brake and Lambert demonstrated how important estrogen is in the development and malignant progression of cervical cancer (94). Low levels of GPER were found to improve overall and recurrence-free survival in the early stage of cervical cancer (95). In addition to the classical ERK1/2 pathway, G-1 was found to downregulate cyclin B in a time-dependent manner in Hela and SiHa cells (96). Di-(2-ethylhexyl) phthalate (DEHP), an environmental xenoestrogen, was also found to trigger the proliferation of cervical cancer cells via activation of the GPER/Akt signaling pathway. The GPER antagonist G-15 was further confirmed to reverse DEHP-induced phosphorylation of Akt in HeLa cells (97).

### Endometrial Cancer

In developed countries, endometrial cancer is one of the most common gynecological tumors and its incidence has increased in recent years (98). The siRNA knockdown of GPER was found to impair cancer invasion and tumorigenesis in RL95-2 cells (99). These findings underscore the role of GPER in cell proliferation and invasiveness of endometrial cancer. In the endometrial cancer cell line KLE (estrogen receptor negative), G-1 was found to be capable of activating the MAPK/ERK pathway and inducing IL-6 secretion (100). Clinical data also suggested high GPER expression to be associated with lower overall survival. The correlation of GPER is stronger than with classical markers, such as ER and EGFR. However, other markers are not significantly related to the overall survival of endometrial cancer patients in the early stage (stage I/II) of this condition (101).

### Prostate Cancer

The function of GPER is not only limited to gynecological cancers; several studies have evaluated the role of this receptor in cancers of the male reproductive system. Graeme Williams et al. reported that modulation of aromatase levels by GPER regulates testosterone and estrogen balance (102). Moreover, G-1 was found to inhibit growth of BPH-1, an immortalized benign prostatic epithelial cell line. Antagonism and knockdown of GPER were both found to inhibit GPER-mediated ERK1/2 activation in prostate cancer cells (103). In addition, benign prostate epithelial cells were noted to possess strong GPER immunoreactivity, indicating that GPER expression is inversely correlated to the degree of neoplastic cell differentiation (104).

High level of GPER was also found in early-stage human prostate stem-progenitor cells indicating GPER as a pathogenesis gene for prostate cancer (105). In addition, BPA could trigger the rapid phosphorylation of p-Akt and p-Erk through GPER to keep the stem-progenitor prostate cells self-renewal and stem cell-like properties in a dose-dependent manner and indicate the prostate cancer risk may increase once exposure to BPA during the development (106). However, Chan et al. work's suggested GPER is a tumor suppressor. Based on G-1 activation, GPER strongly suppressed PC-3 cell proliferation in both *in vitro* and *in vivo* assays. The cells arrested at the G2 phase and phosphorylation of G2-checkpoint regulators NF-YA were also observed after activation (107). In LNCaP xenografts model, their G-1 mediated GPER activation could suppress tumor formation in both the androgen-sensitive (AS) and the castration-resistant (CR) stage. However, the effect was not significant if the animal was in high testosterone status (108). This result also indicates the potential cross-talk between the androgen receptor and GPER.

### Thyroid Cancer

The large gender difference in thyroid cancer incidence rates underscores that estrogen and related pathways are involved in its tumorigenesis (109). Ping et al. studied cadmium-induced thyroid cancer in WRO and FRO cell lines (61). Results suggested that GPER enhances the response to cadmium, greatly influencing cellular proliferation and migration via activation of the ERK, NF-κB, and Akt pathways. Another study concerning thyroid papillary cancer evaluating BHP10-3 cells also reported that the GPER antagonists ICI 182,780 (fulvestrant) and G-15 significantly suppressed thyroid cancer progression (110).

### Lung Cancer

Estrogen and its receptors have been proposed to serve major roles in the progression and metastasis of lung cancer. Their functions are indeed deeply involved with EGFR interactions (111). Avino et al. clarified the interaction between IGF-1, DDR1 and GPER. DDR1 and GPER are both crucial to IGF-1-stimulated chemotactic motility in mesothelioma and lung cancer cells; this function is dependent on the formation of IGF-1-DDR1-GPER complexes (59). In addition, cytoplasmic GPER expression was found to correlate to more advanced cancer stages (IIIA–IV), lymph node metastasis, and poor differentiation in non-small cell lung cancers (112). Furthermore, G-1 was reported to induce malignant cell proliferation, invasion, and migration in primary cultured cancer cells.

### Other Cancers

Due to the ubiquitous expression of GPER, it is highly likely that this receptor is involved in a variety of malignancies. A study investigating adrenocortical carcinoma found that GPER agonism exerts an inhibitory effect on H295R cell growth (113). The function of GPER in adrenocortical carcinoma has been proposed to involve mitochondria-related signaling, in which GPER, via the EGR-1 pathway, positively regulates the mitochondrial apoptotic pathway via B-cell lymphoma-2-Associated X (BAX) thereby inhibiting tumor growth (114). In bladder cancers, GPER was reported to counteract E2-stimulated cell proliferation in T24 carcinoma cells (115).

In summary, the above data from cancer cell lines suggested the potential correlation the GPER in cancer progression and metastasis. However, as mentioned in breast cancer study, there is no significant difference in breast cancer tumorigenesis in GPER knock-out mice when compared to wild-type animals (116). This makes the conclusion still under debate. Even the role of GPER may be involved in cell proliferation, cell migration, or tumor microenvironment, but there is a lack of *in viv*o data and molecular mechanism of those effects. Therefore, more researches by using different cancer animal models and further analysis of patient samples are required to validate the role of GPER in cancers.

### Perspective

As hormonal therapy is widely used in the management of a variety of cancers, the roles GPER plays in malignancies warrants continued study. GPER indeed plays an important role in cancers of both male and female reproductive systems. Although tamoxifen and fulvestrant are widely used ER antagonists in hormonal therapy, their response rates among breast cancer patients remain relatively low, even in ER-positive patients (117). As both tamoxifen and fulvestrant are GPER agonists, the activation of this receptor and its downstream signaling pathway likely facilitates drug resistance among ER-positive patients that express high levels of GPER.

When we discuss the role of GPER, we should also aware of the potential role of membrane ERs in cell signaling. There is increasing evidence suggested the important role of plasma membrane ERs in triggering the membrane-initiated steroid signaling (MISS) that regulating multiple physiological events. The membrane localization of ERα is depended on the palmitoylation at C451 (C447 in humans) (116). Thus, the palmitoylation-loss ERα transgenic mice (C451A-ERa, also called nuclear-only ERα, NOER) served as the key animal model and provided solid evidence in studying the role of MISS. The loss of membrane localization caused the C451-ERα female mice suffering serious abnormality in ovaries and infertility. This mutation also impaired the E2-dependent vascular function, including vasodilation and endothelial repair (118). A similar conclusion was found in another research: infertility, abnormal ovaries, hypoplastic uteri, and impairer mammary gland ductal development were identified in the transgenic mice with the same mutant (C451A) (119). In males, significant reduction of epididymal sperm motility and abnormalities of sperm was observed in C451-ERα mice (120). But it is interesting to note that in Adlanmerini et al. study, the effect of E2 on uterus, endometrial epithelial proliferation, was unaffected in C451-ERα mice. This indicates the membrane signaling of estrogen is tissue-dependent (118). Indeed, the study of membrane ERα in other estrogen responsive tissues suggested this tissue-dependent MISS effect, for example, estrogen response to trabecular bone mass is strongly dependent on MISS, while the liver weight and total body fat mass only slightly affect in C451-ERa mice. MISS also involved in cancer cell proliferation. In which the MISS of ERα mediate the proliferative effects, while ERβ could trigger the anti-proliferative effects of E2 (121). Therefore, GPER is functioning as a parallel or alternative pathway of E2. Depends on cell types, it could be a dominant pathway in ERs negative cells. Even this review is focused on GPER, the role of ERs MISS, other steroid receptors and their potential cross-talk with GPER should be considered and further investigation is required to verify this important concept in estrogen research.

Now, we understand that when GPER combined with the classic ER at the cellular level, it may act either in synergistically or antagonize with each other, with the ultimate cellular output being dependent on the integration of all the stimulated and inhibited pathways. However, we should consider more possible aspects that GPER also participates in canonical and non-canonical GPCR transduction pathways, which may work as alternative signaling in cells without expressing both androgen receptor and ERs. Such examples could be found mainly in triple-negative breast cancer or after-treat prostate cancer. As androgen is known to reduce GPER expression by androgen receptor, the AR knock-out may further enhance the GPER mediated signals. Although the detailed replacing signaling has not been clearly demonstrated, the promising role for GPER as a therapeutic target has already emerged. Therefore, despite the accumulating findings, detailed GPER functions are still quite opaque. First of all, it is notable that in currently accepted models are quite difficult to completely eliminate the ER effect with GPER. Secondly, the complex cross-talk between GPER and the non-genomic actions of other sex-steroid also lead to inconsistent results between publications. Last but not least, the involvement of other sex-related steroids also makes the situation complicated. But based on the advance of technologies, such as the new genome-editing method and the development of highly selective and affinity agonist and antagonist for GPER, the investigation of GPER could be intensively studied to unify the rapid hormonal response between GPER and other steroid receptors.

**Figure 1:**
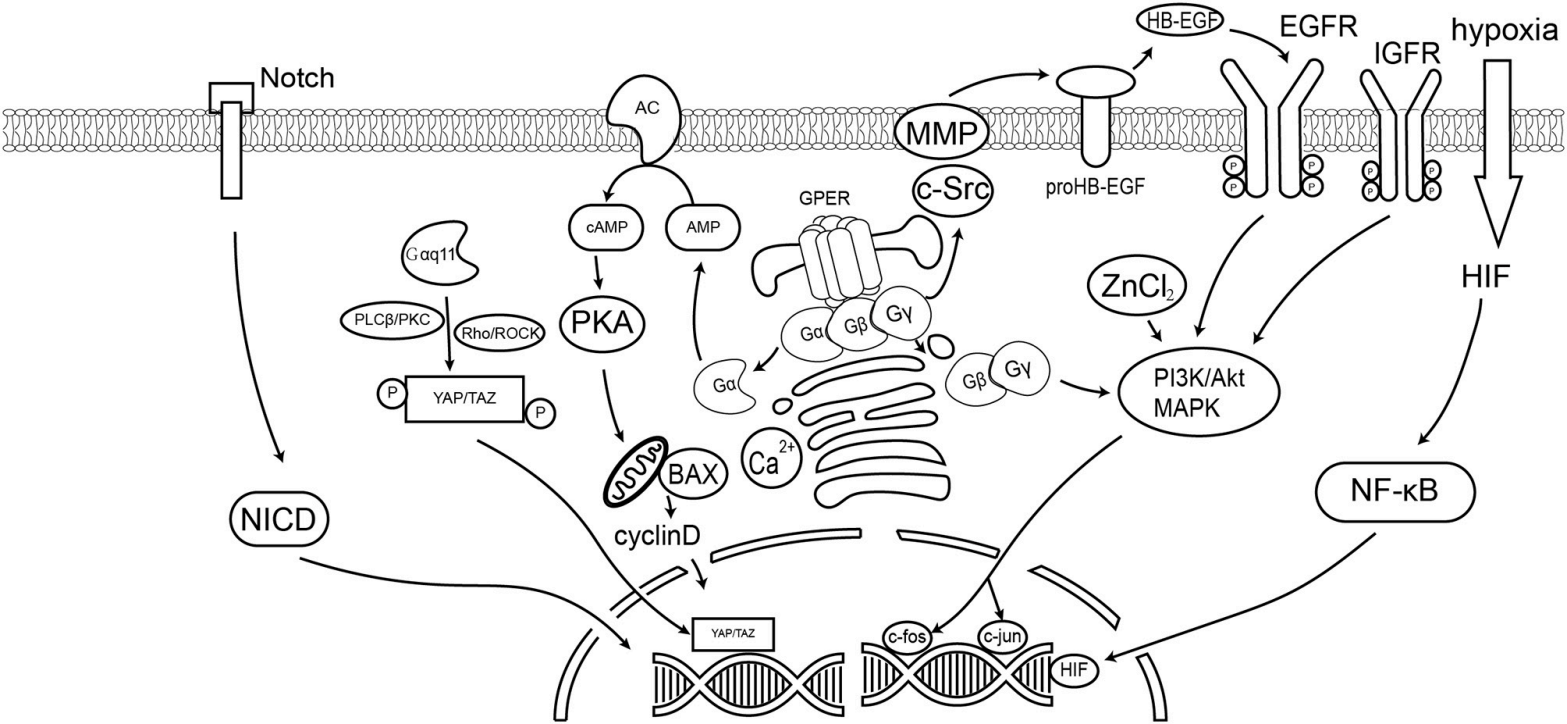
GPER signaling pathways in cancer cells. This diagram summarizes the signaling events discussed in this article. GPER mediated the non-genomic effect of E2 and active the G-protein dependent signaling, including the cAMP, Ca^2+^, Akt, and YAP/TAZ pathways. GPER could also trigger the ligand-dependent transactivation of receptor tyrosine kinase, such as EGFR. Previous reports also suggest GPER could affect the Notch signaling and hypoxia stimulated HIF pathway.

A number of studies have reported that GPER mediates multiple signaling pathways. Those molecular pathways are summarized in Table 1 and Figure 1. Within those pathways, the activation of ERK1/2 is undoubtedly the pathway most consistent across cell types. Activation of the ERK1/2 pathway has likewise been suggested to be the key factor in cancer prognosis (107). Thus, ER-targeting drug resistance likely occurs due to GPER pathway activation. Therefore, GPER modulation is a potential novel strategy in cancer therapy. Of them, G-15 and G-36 are widely used in cancer study, but there remains a lack of solid clinical evidence supporting their specificity. Apart from small molecule inhibitors, siRNA are also potential options for gene-specific inhibition. However, delivery remains the major limitation of siRNA drug application. Recent advances in nanotechnology, however, have improved our understanding concerning the use of nanoparticles as novel non-viral delivery methods. Both *in vitro* mechanistic studies and *in vivo* therapeutic validation have been reported. Hence, direct regulation of GPER expression by nanotechnology offers a novel and efficient tool for anticancer therapy and warrants further investigative efforts.

**Table 1:** Structure and functions of GPER agonists and antagonists.

**Name**	**Structure**	**IC_**50**_/EC_**50**_ and functions**	**References**
**AGONIST**			
G-1	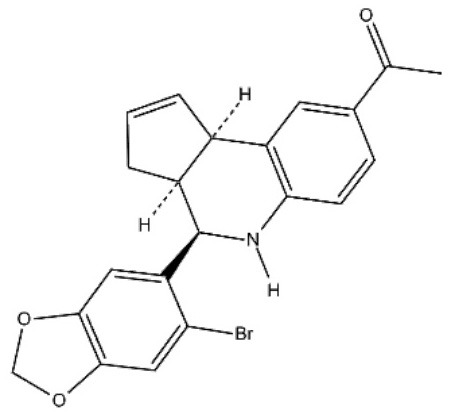	Anti-proliferative activities: 0.54 (SK-BR-3) 39.92 (MCF-7)	(107)
IC182,780 (Fulvestrant)	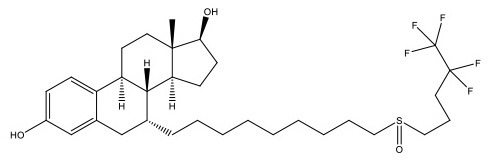	Direct killing cancer cells 35 μCi (MCF-7 cell xenografts)	(122)
5408-0877	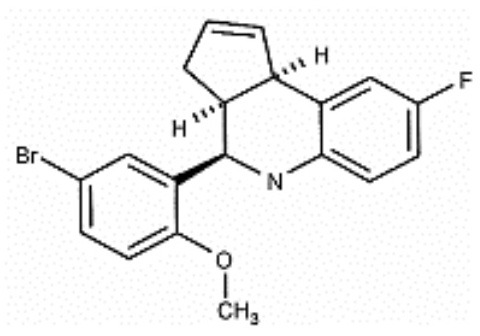	Relaxation of carotid arteries in Sprague-Dawley rats IC50 value = 10 μM	(123)
**ANTAGONIST**			
G-15	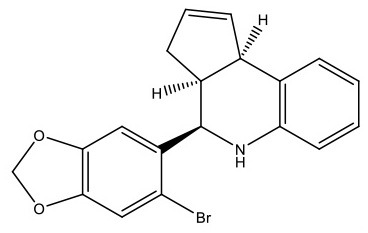	dose-dependent cytotoxicity IC50 value 11.2 μM for SCC4, 15.6 μM for SCC9, 7.8 μM for HSC-3	(124)
G-36	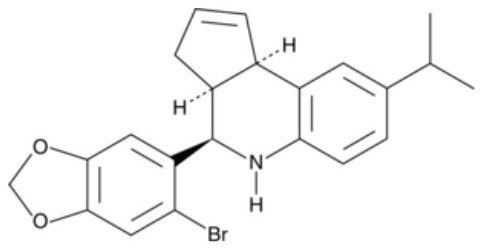	G15 abolished the proliferative effects at IC50 = 112 nM	(125)
C4PY (*meso*-(*p*-acetamidophenyl)-calix[4]pyrrole)	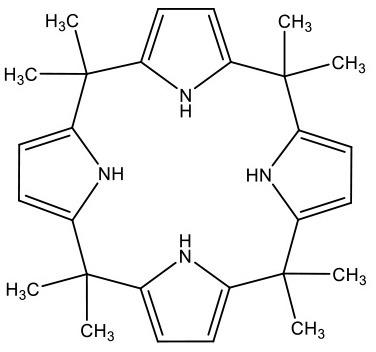	cytotoxicity EC50 <5 μM when tested on A549 and H727 cell lines	(126)

## Author Contributions

All authors listed have made a substantial and direct input in preparing this manuscript, and approved it for publication.

### Conflict of Interest

The authors declare that the research was conducted in the absence of any commercial or financial relationships that could be construed as a potential conflict of interest.
